# Effectiveness of Psychosocial Interventions on Stress, Anxiety, Depression, and Quality of Life in Parents of Children, Adolescents, and Young Adults With Cancer: A Meta‐Analysis of RCTs

**DOI:** 10.1111/nhs.70156

**Published:** 2025-06-10

**Authors:** Cigdem Sari Ozturk, Emine Gunes San, Sumeyye Yildiz

**Affiliations:** ^1^ Pediatric Nursing Department Gazi University Nursing Faculty Ankara Turkey; ^2^ Faculty of Health Sciences, Department of Nursing Bartin University Bartin Turkey

**Keywords:** anxiety, depression, meta‐analysis, neoplasms, parents, psychology, quality of life

## Abstract

The meta‐analysis aimed to investigate the effectiveness of psychosocial interventions applied to parents of children, adolescents, and young adults undergoing cancer treatment. PubMed, Cochrane Library, MEDLINE, ProQuest, Science Direct, Scopus, and Web of Science were searched to identify eligible randomized controlled trials from January 1, 2013 and April 2024. Search was limited to “parents of children, adolescent, and young adults” and “psychosocial interventions.” Of 9327 articles, 21 articles met the inclusion criteria. The revised Cochrane risk‐of‐bias tool for randomized trials (Rob2) was used. In all, 1926 parents of children with cancer were included. PROSPERO number is CRD42023446776. Psychosocial interventions demonstrated significant effects across various outcomes based on Hedge's *g*. They showed a moderate effect on anxiety (*g* = −0.538, 95% CI: [−0.941, −0.135]), a large effect on depression (*g* = −0.834, 95% CI: [−1.328, −0.339]), a large effect on the quality of life (*g* = 1.375, 95% CI: [0.367, 2.382], and a moderate effect on stress (*g* = −0.798, 95% CI: [−1.393, −0.203]). Consequently, psychosocial interventions applied to parents have significant effects on reducing anxiety, depression, and stress and improving the quality of life.

**Summary**

- Psychosocial interventions, including cognitive behavioral therapy techniques (e.g., emotion regulation, resilience programs, and stress management) and other methods (e.g., problem‐solving therapy, psychosocial education, and counseling), are effective in reducing anxiety, depression, and stress levels while improving the quality of life for parents of children with cancer.
- No significant difference was observed in the effect sizes of cognitive behavioral therapies and other psychosocial methods on anxiety, depression, stress, and quality of life, suggesting that both types of interventions are equally beneficial and can be tailored to parents' individual needs and preferences.
- Integrating structured, flexible, and culturally sensitive psychosocial programs into healthcare systems is essential to support parents' emotional and psychological well‐being during their child's cancer treatment.

## 1 Introduction

Childhood cancers are an important and current public health problem that is increasing worldwide. More than 300,000 children worldwide are diagnosed with cancer every year, and one child dies from cancer every 3 min (World Health Organization 2021). The most common types of cancer in children aged 0–14 years are leukemia (75%), brain and other central nervous system tumors, and lymphomas. While cancer rates are lower in children than in adults, survival rates are significantly higher, reaching 84% on average in high‐income countries (American Cancer Society 2017; Cancer Research 2016; World Health Organization 2021). The comparatively higher survival rates of children with cancer have led to the examination of the effects of the treatment and care process on the quality of life and family processes of children and their families (Ozkul and Partlak Gunusen 2019).

Childhood cancer is a serious disease that imposes significant emotional, financial, and practical challenges on families. The treatment and ongoing care of children with cancer can be a long and stressful journey for parents, often leading to emotional distress, financial strain, and difficulties in accessing healthcare services (Walubita et al. 2018). Parents frequently experience psychosocial problems, including anxiety, depression, and posttraumatic stress symptoms (PTSD), which can also influence their children's emotional and behavioral well‐being (Koutná and Blatný 2020). A meta‐analysis found that the prevalence of anxiety, depression, and PTSD among parents of children with cancer is significantly higher than in the general population (van Warmerdam et al. 2019).

Given these challenges, providing psychosocial support for parents is crucial to ensuring the well‐being of the entire family (Christen et al. 2019). Psychosocial interventions such as cognitive behavioral therapy (CBT), problem‐solving skills training (PSST), and family therapy have been shown to reduce parents' stress and improve coping skills. For example, CBT focuses on modifying environmental and behavioral factors contributing to emotional distress, while PSST helps parents develop practical strategies to address challenges effectively (Eccleston et al. 2015; Koumarianou et al. 2021). Such interventions can also indirectly benefit children's treatment outcomes by fostering a supportive family environment (Ljungman et al. 2018; Pouraboli et al. 2019).

Integrating early and ongoing psychosocial interventions into pediatric oncology care is essential. Various psychosocial intervention programs have been implemented and evaluated through meta‐analysis studies (Koumarianou et al. 2021; Sheng et al. 2023). These programs are generally classified into two categories: (1) cognitive behavioral therapies, including emotion regulation, logotherapy, communication skills, positive thinking, brief therapy, and relaxation–resilience programs, and (2) other psychosocial interventions, such as problem solving, psychosocial training, and counseling (Koumarianou et al. 2021; Sheng et al. 2023). These programs must be accessible, relevant to families' needs, and culturally sensitive to maximize their impact (Kearney et al. 2015). By addressing the challenges parents face during their child's treatment, these interventions can improve both parent and child well‐being (Koumarianou et al. 2021).

A review of literature highlights the diversity of psychosocial programs aimed at supporting parents of children with cancer (Eche et al. 2021; Luo et al. 2021a). Luo et al. (2021a) conducted a randomized controlled trial demonstrating that resilience training, self‐disclosure, and peer support interventions enhance parental resilience (Luo et al. 2021a). Additionally, studies incorporating problem‐solving training, psychoeducational therapy, cognitive behavioral approaches, family therapy, stress management, and coping development therapy have demonstrated significant reductions in parental anxiety and depression (Eche et al. 2021). On the other hand, no meta‐analysis of randomized controlled studies on parents of children receiving cancer treatment could be found. Therefore, it is thought that this meta‐analysis will provide important evidence to demonstrate the effectiveness of psychosocial interventions applied to parents of children, adolescents, and young adults undergoing cancer treatment.

This study is based on the last decade of research, which has seen significant advances in research methods, technological developments, and the increased adoption of interdisciplinary approaches. Furthermore, some older studies may be methodologically inadequate by today's standards or may contradict current findings. Therefore, focusing on the last decade of research allows the study to reflect current knowledge and obtain more accurate results. Moreover, the time under consideration serves to enhance the validity and applicability of the study by encompassing the most recent trends and developments in the field. This meta‐analysis aims to evaluate the evidence from RCTs on the effects of psychosocial interventions for parents of children with cancer on anxiety, depression, stress, and quality of life. Only RCTs were included to ensure that high‐quality studies were included.

To best address the research problem, our research questions consisted of the following:

- What is the effect of psychosocial interventions on the management of anxiety, depression, stress, and quality of life among parents of children, adolescents, and young adults with cancer?
- Which type of psychosocial interventions are more effective for parents of children, adolescents, and young adults with cancer? Is the type of psychosocial intervention a moderator variable?
- What is the effect size of psychosocial interventions according to the duration of the interventions?

## 2 Methods

### 2.1 Search Strategy

This meta‐analysis was reported using guide by the Preferred Reporting Items for Systematic Reviews and Meta‐Analysis (PRISMA) 2020 checklist, PRISMA 2020 abstract checklist (see File S1), and PRISMA flow diagram. The study protocol was registered in the International Prospective Register of Systematic Review (PROSPERO) database [CRD42023446776]. A search that covered records from January 1, 2013 to April 2024 was conducted on seven databases, including PubMed, Cochrane Library (Cochrane Central Register of Controlled Trials), MEDLINE, ProQuest, Science Direct, Scopus, and Web of Science. We also manually searched the reference list of all identified articles to identify additional studies. Keywords used for the search were “child*,” “pediatric,” “adolescent,” “young,” “adult,” “cancer,” “neoplasm”, “oncology,” “family,” “parents,” “caregivers,” “intervention*,” “prevent,” “social,” “psycho*,” “support.” The search strategy was adapted to each database. To ensure the comprehensiveness of our search, we used a combination of both general terms (e.g., “psychosocial,” “psychological”) and specific intervention related terms (e.g., “cognitive behavioral therapy,” “CBT,” “resilience,” “coping,” “stress management,” and “problem‐solving therapy”). Boolean operators and database‐specific syntax were used to increase the sensitivity of the search across all platforms. The full list of search terms and strategies is provided in File S2.

### 2.2 Eligibility Criteria

The PICOS (Population, Intervention, Comparison, Outcomes, and Study Design) approach created the study protocol questions. The inclusion and exclusion criteria are as follows:

#### 2.2.1 Studies are Eligible if They

P—Population: Parents of children, adolescents, and young adults undergoing cancer treatment. We included only studies in which the participating caregivers were explicitly described as parents. Studies involving other types of family members, such as grandparents or siblings, were excluded.

I—Intervention: Psychosocial interventions (including cognitive behavioral therapy [CBT]‐based techniques) (e.g., emotion regulation, logotherapy, positive thinking, communication skills training, stress management, resilience programs, relaxation programs, solution focused therapies, brief therapy, and direct cognitive behavioral therapy techniques) and other psychosocial methods (e.g., filial therapy, problem‐solving therapy, animal‐assisted intervention, and psychosocial education and counseling interventions). Studies that included combination therapies were eligible if a structured psychosocial intervention constituted a clearly defined and central component of the treatment.

C—Comparison: active or passive condition.

O—Outcomes: anxiety, depression, stress, and quality‐of‐life levels.

S—Study design: Randomized controlled studies.

The exclusion criteria were as follows:

- Systematic reviews
- Nonrandomized controlled trials
- Studies that focused only on child and/or siblings‐centered psychosocial interventions
- Studies that did not focus on parent outcomes (state anxiety, depression, stress, or quality of life)
- Studies including insufficient numeric data for analysis

### 2.3 Data Extraction

EndNote 20 software (Thomson Corporation) was used to record duplicate studies in our study. Two authors (EGS and SY) independently screened all articles by abstract and title using inclusion/exclusion criteria. Disagreements about inclusion were resolved through discussion. In cases where consensus could not be reached and disagreements emerged, the opinion of the third author (CSO) was used to achieve consensus. This study used a data coding form created by reviewing the literature (Ebrahimi et al. 2019; Eche et al. 2021). The coding included the study's author, year of publication, type of study, design, sample size, type of intervention, type, duration, measurement times, measurement tools, and findings. For studies involving combination therapies, categorization was based on the dominant psychosocial content described in the methodology section of the original articles. Each intervention was assigned to the CBT‐based or other psychosocial methods group depending on its primary theoretical orientation and emphasized techniques. The reliability of the coded data was obtained from comparisons made by three researchers (CSO, EGS, SY).

### 2.4 Assessment of Bias in Study Inclusion

**FIGURE 1:**
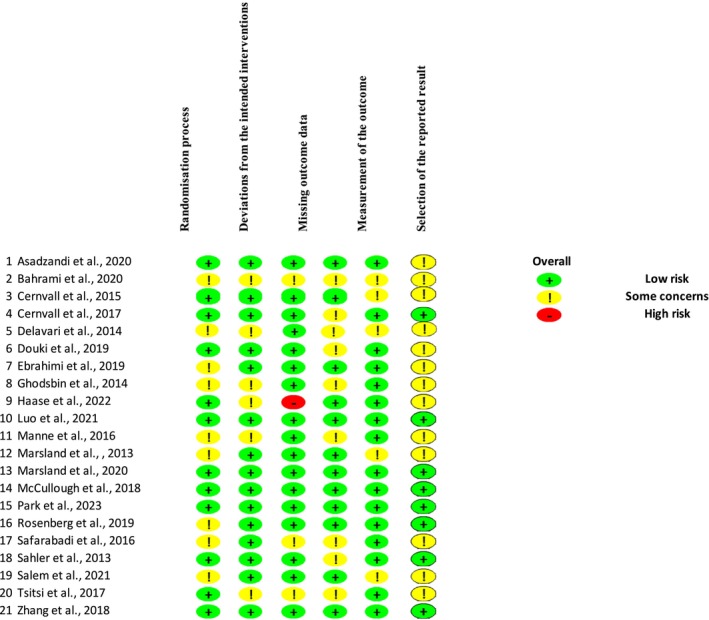
Risk of bias assessment for studies.

The risk bias of included studies was assessed using the revised Cochrane risk‐of‐bias tool for randomized trials (RoB 2) (Higgins et al. 2019). The tool assesses the risk of bias via five criteria: (1) bias arising from the randomization process; (2) bias due to deviations from intended interventions; (3) bias due to missing outcome data; (4) bias in the measurement of the outcome; and (5) bias in the selection of the reported result by the Cochrane handbook. Risk of bias assessment of the included studies (*n* = 21) was performed with RoB 2 (Figure 1). The risk of bias assessment was conducted using the revised Cochrane risk‐of‐bias tool for randomized trials (RoB 2) (Higgins et al. 2019). Two authors (EGS and SY) independently appraised the articles quality by RoB2. Each domain of RoB2 is evaluated at three levels: low risk, some concerns, and high risk. Each item is assessed as “yes” or “no” or “partly yes.” Any disagreements between the reviewers regarding the assessment of study quality were resolved through discussion or by consulting a third author. When the results were combined, a common result pattern was created by the RoB2 checklist (Higgins et al. 2019).

### 2.5 Analytical Strategy

In this meta‐analysis, the Comprehensive Meta‐Analysis software (CMA) version 2.0 was used (Borenstein et al. 2005). The primary outcomes of the meta‐analysis are anxiety, depression, quality of life, and stress. “Hedges' *g*” was used to calculate the effect size of continuous data based on means and standard deviations for the intervention and control group (between‐group) postintervention. Additionally, if there are studies with small samples in the meta‐analysis study, it is recommended to use Hedges' *g* (Dincer 2019). Hedges' *g* is similar to Cohen's *d* and may be interpreted using the same grades as small (0.2), medium (0.5), and large (0.8). Heterogeneity was assessed using Cochran's *Q* test (*p* < 0.10) and the *I* ^2^ statistic. A significant *Q* test (*p* < 0.10) indicates heterogeneity beyond random variation. Since *Q* test has low power with few studies, *I* ^2^ was used to quantify heterogeneity as low (≤ 25%), moderate (~50%), or high (≥ 75%) (Cohen 1992). Considering the heterogeneity in this study, a random effects model was used. (Borenstein's 2009) recommendation to choose a random effects model for articles collected from the literature was also considered in this study. This study assessed publication bias using Egger's test, Begg and Mazumdar rank correlation, Rosenthal and Orwin fail‐safe N, and Funnel plot analysis (Borenstein et al. 2021). Egger's test evaluates funnel plot asymmetry, where a statistically significant *p* value (< 0.05) indicates potential publication bias, while a nonsignificant result suggests bias is unlikely. Begg and Mazumdar rank correlation further assesses asymmetry, and a nonsignificant result (*p* > 0.05) supports the absence of bias. Additionally, Rosenthal and Orwin's fail‐safe N estimates how many missing studies with null effects would be required to render the observed effect size nonsignificant, with a high fail‐safe N indicating robust findings. Funnel plots for the outcomes of the study are included in File S3. In the study, the significance level of the statistical analyses was taken as 0.05. Metaregression and subgroup analyses were performed to determine potential factors affecting the results of the interventions (Higgins and Green 2011). ANOVA was performed according to the type of intervention, and metaregression analysis was performed according to the duration of the intervention.

In the meta‐analysis study, PROSPERO recording was performed before the start of the study. Registration number: CRD42023446776. Since the study was a meta‐analysis, ethics committee approval was not required in accordance with standard practice.

## 3 Results

### 3.1 Selection of Studies

**FIGURE 2:**
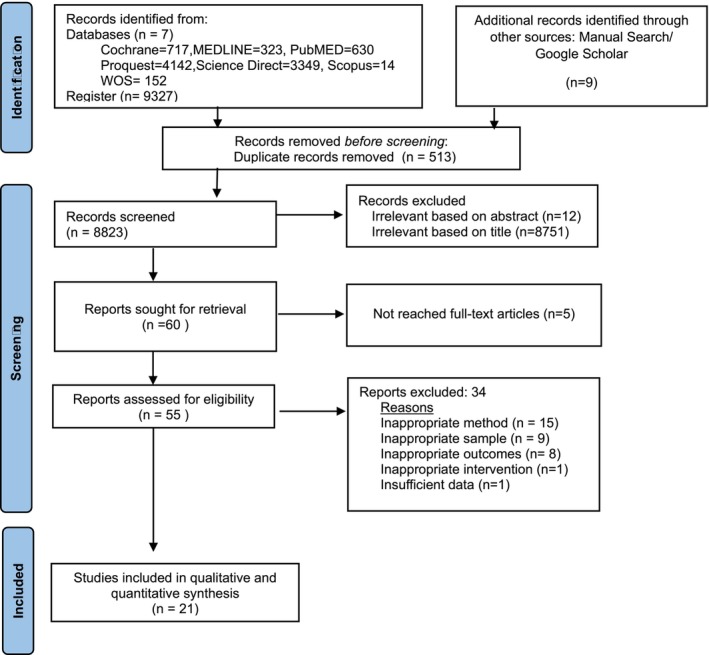
Flow chart of the study selection process according to the Preferred Reporting Items for Systematic Reviews and Meta‐Analyses (PRISMA).

In total, 9327 studies were found in the databases. After removing duplicates, 8823 results were reviewed based only on the title. Subsequently, 55 studies were analyzed based on titles and abstracts, and 34 were excluded. The selection of studies included in the analysis is detailed in the PRISMA flowchart in Figure 2, and the reasons for the exclusion of studies are provided in File S4. Finally, 21 studies were included.

### 3.2 Characteristics of the Study

Some details of the included studies are presented in Table 1. The study sample size ranged from 15 to 157. Of a total population of 1926 participants, 986 were in the intervention group, and 940 were in the control group. In this meta‐analysis, psychosocial intervention techniques were divided into cognitive behavioral therapy techniques and other methods in line with the literature (Koumarianou et al. 2021; Sheng et al. 2023). Emotion regulation (Bahrami et al. 2020), logotherapy (Delavari et al. 2014), positive thinking (Douki et al. 2019; Zhang et al. 2019), communication skills (Haase et al. 2022), stress management (Rosenberg et al. 2019; Marsland et al. 2013, 2020), resilience programs (Cernvall et al. 2015), relaxation programs (Tsitsi et al. 2017), solution focused therapies (Zhang et al. 2018), and direct cognitive behavioral therapy techniques (Haase et al. 2022; Manne et al. 2016; Park et al. 2023; Salem et al. 2021) are grouped as cognitive behavioral therapy techniques in line with the literature (Eche et al. 2021; Luo et al. 2021b). This study grouped psychosocial interventions other than cognitive behavioral therapy techniques as other methods. Other methods included filial therapy (Ebrahimi et al. 2019), animal assisted intervention (McCullough et al. 2018), problem‐solving therapy (Sahler et al. 2013), and psychosocial education and counseling interventions (Asadzandi et al. 2020; Ghodsbin et al. 2014; Luo et al. 2021a; Safarabadi‐Farahani et al. 2016).

**TABLE 1:** Study characteristics.

Author	Country	Sample size and age (years)	Intervention delivery method	Intervention type	Intervention duration	Outcomes	Measurement times	Instruments	Results
Asadzandi et al. (2020)	Iran	36/36 Age = 35–36	Spiritual counseling Face to face	Other methods	16 weeks	Anxiety Depression Stress	t1: Baseline t2: 16 weeks	Depression, Anxiety, Stress Scale (DASS 21)	There was a statistically significant difference in the mean scores of depression (*p* < 0.001), stress (*p* = 0.003), and anxiety (*p* < 0.001) between the two groups after the intervention.
Bahrami et al. (2020)	Iran	30/30 Age = 20–55	Emotion regulation training Face to face	Cognitive behavioral therapy	8 weeks	Anxiety	t1: Baseline t2: 8 weeks t3: 12 weeks t4: 16 weeks t5: 20 weeks	Beck Anxiety Inventory	The repeated measures ANOVA revealed a significant difference between the two groups in terms of the effect of the intervention (*p* < 0.001).
Cernvall et al. (2015)	Sweden	31/27 Age = 36–40	Online Face to face	Cognitive behavioral therapy	10 weeks	Anxiety Depression Stress	t1: Baseline t2: 10 weeks	Beck Depression Inventory Beck Anxiety Inventory	There was a significant intervention effect on depression and anxiety and reductions in the intervention group with Large within‐group effect sizes (*d* = 0.85–1.09).
Cernvall et al. (2017)	Sweeden	31/27 Age = 36–40	Face to face	Cognitive behavioral therapy	12 weeks	Anxiety Depression Stress	t1: Baseline t2: 10 weeks	PTSD Checklist Civilian Version = Stress Beck Depression Inventory Beck Anxiety Inventory	Intention‐to‐treat analyses revealed significant effects in favor of the intervention on the primary outcome PTSS, with large between‐group effect sizes at postassessment (*d* = 0.89; 95% CI 0.35, 1.43) and at 12‐month follow‐up (*d* = 0.78; 95% CI 0.25, 1.32). Significant effects in favor of the intervention on the secondary outcomes of depression and anxiety were also observed.
Delavari et al. (2014)	Iran	15/15 Age = 25–40	Logotherapy Face to face	Cognitive behavioral therapy	10 weeks	Anxiety Depression	t1: Baseline t2: 10 weeks	Beck Depression Inventory (BDI) Beck Anxiety Inventory (BAI)	Logo therapy has a significant effect in reducing anxiety and depression among mothers of children with cancer (*p* < 0.05)
Douki et al. (2019)	Iran	15/15 Age = 18–60	Positive thinking training Face to face	Cognitive behavioral therapy	8 weeks	Anxiety, Depression Quality of Life	t1: Baseline t2: 8 weeks	DASS questionnaire The MOS	The mean scores of depression and anxiety respected, in the intervention group were significantly lower than the control group (*p* < 0.001) and (*p* < 0.004). Furthermore, significant differences were found between the study groups in quality‐of‐life scores (*p* < 0.05)
Ebrahimi et al. (2019)	Iran	16/16 Age = 36–39	Filial therapy Face to face	Other methods	10 weeks	Anxiety Depression Stress	t1: Baseline t2: 10 weeks	Depression, Anxiety and Stress Scale (DASS)	Mothers in the filial therapy group experienced a significant decrease in their level of depression, anxiety, and stress in the posttest (*p* < 0.001).
Ghodsbin et al. (2014)	Iran	40/40 Mothers =34.1–34.8 Fathers = 44.6–46.8	Educational program Face to face	Other methods	12 weeks	Quality of life	t0: Baseline t1: 12 weeks	QoL (Quality of Life of Bone Marrow Transplant: QOL‐BMT)	After 3 months changed to 338.2 and 226.7, respectively. *T* test verified these increases (*p* < 0.05) in quality of life in the intervention group.
Haase et al. (2022)	USA	56/54 Age = 28–62	Self‐care and Communication intervention face to face	Cognitive behavioral therapy	6–8 week	Anxiety Stress Quality of Life	t0: Baseline t1: 2 weeks postintervention t3: 12‐week postintervention	Spielberger State–Trait Anxiety Inventory–State Component, Perceived Stress Scale	Both groups exhibited significant within‐group improvement for parent distress (state anxiety, T3; perceived stress, T2 and T3; mood, T3), state anxiety (T2) intervention only, and family strengths control group only.
Luo et al. (2021a)	China	52/51 Age = 35–37	Educational intervention Face to face	Other methods	8 weeks	Depression Quality of Life	t0: Baseline t1: 8 weeks t3: 32 weeks	The Self‐Rating Depression Scale The Short Form of the 6‐Dimension Health Survey	The participants The experimental group showed significantly higher levels of resilience (mean 67.96, SD 15.8 vs. mean 58.27, SD 19.0; *p* < 0.001) and lower levels of depressive symptoms (mean 40.17, SD 9.9 vs. mean 46.04, SD 10.9; *P* < 0.001) than those in the control group. At 6 months of follow‐up.
Manne et al. (2016)	USA	*n* = 218 Group 1: (*n* = 108) Group 2: (*n* = 110) Age = 37–38	Social‐cognitive processing intervention Face to face and phone	Cognitive behavior therapy	14 weeks	Anxiety Depression Stress	t0: Baseline t1: 4 weeks t3: 24 weeks t4: 48 weeks	Beck Depression Inventory Impact of Event Scale = Distress Beck Anxiety Inventory	SCIP reduced the caregiver's distress significantly more than BPC between the pretransplant assessment (Time 1) and 1‐month follow‐up assessment (Time 2).
Marsland et al. (2013)	USA	23/14 Age = 28–73	Stress management intervention Face to face Six telephone contacts, and Access to a study website	Cognitive behavior therapy	16–24 weeks	Anxiety Depression Stress	t0: Baseline t1: 24 weeks	Anxiety Questionnaire (MAQ) Beck Depression Inventory State–Trait Anxiety Inventory Perceived Stress Scale	There was no overall significant impact of participation in the intervention on levels of distress at T2.
Marsland et al. (2020)	USA	60/60 Age = 19–57	Stress management intervention Face to face	Cognitive behavior therapy	20–24 weeks	Anxiety Depression Stress	t0: Baseline t1: 24 weeks t3: 48 weeks	The Beck Depression Inventory (BDI) The State–Trait Anxiety Inventory (STAI) The Perceived Stress Scale (PSS)	Enrolment, retention, and satisfaction data supported feasibility and acceptability. Latent change score models showed the intervention reduced perceived stress (*d* = −0.37, *p* = 0.03), anxiety symptoms (*d*s = −0.38 and −0.56, *p*s < 0.03), and a nonsignificant effect for depressive symptoms (*d* = −0.29, *p* = 0.11)
McCullough et al. (2018)	USA	60/46 Age = 18–50	Animal‐assisted intervention Face to face	Other methods	Over 16 weeks	Anxiety Stress Quality of Life	t0: Baseline t1: 16 weeks	State–Trait Anxiety Inventory Pediatric Quality of Life Inventory Pediatric Inventory for Parents	Parents in the intervention group showed significantly decreased parenting stress (*p* = 0.008), with no changes in stress among parents in the control group.
Park et al. (2023)	South Korea	20/22 Age = 41–43	Internet‐based family resilience promoting program Online	Cognitive behavior therapy	8 weeks	Depression	t0: Baseline t1: 4 weeks t2: 8 weeks	Korean version of the Beck Depression Inventory	However, there was no significant difference between the groups in the level of depression (*β* = 2.133, *p* = 0.187, effect size = 0.416).
Rosenberg et al. (2019)	USA	32, 32/30 Age = 35–38	Promoting resilience in stress management intervention Face to face	Cognitive behavior therapy	12 weeks	Stress Quality of life	t0: Baseline t1: 12 weeks	Perceived Stress Scale Median (SD) Kessler Psychological HRQOL	One‐on‐one PRISM‐P delivery was significantly associated with improvement compared with usual care in parent‐reported outcomes for resilience (β, 2.3; 95% CI 0.1, 4.6; *p* = 0.04) and for benefit finding (β, 0.5; 95% CI 0.2, 0.8; *p* = 0.001).
Safarabadi‐Farahani et al. (2016)	Iran	32/33 Age = 32–34	Psychosocial intervention Face to face and phone	Other methods	5 weeks	Quality of life	t0: Baseline t1: 5 weeks t2: 9 weeks	The Caregiver's Quality of Life Index‐Cancer Persian version	Significant improvement was found within the intervention group on QOL. (*p* < 0.001) including improvements on subscale measures of mental/emotional burden (*p* < 0.001), Disruption (*p* < 0.001), and positive adaptation (*p* < 0.001), compared with the control group over time.
Sahler et al. (2013)	USA	152/157 Age = 36–38	Problem‐solving skills training Face to face	Other	12 weeks	Depression	t0: Baseline t1: 12 weeks t2: 24 weeks	Beck Depression Inventory‐Square Root	Except for the level of problem‐solving skill, which was directly taught in the PSST arm, outcome measures improved equally in both groups immediately after postintervention.
Salem et al. (2021)	Denmark	94/110 Age mean = 36.5	Home‐based cognitive behavioral therapy Face to face	Cognitive behavior therapy	48 weeks	Anxiety Depression Posttraumatic stress	t0: Baseline t1: 24 weeks t2: 48 weeks	PTSD Symptom Checklist‐92‐Revised.	Parents in the intervention group did not show a statistically significant decrease in symptoms of PTSD as compared with The control group at 6 months (predicted mean difference, −0.10; 95% confidence Interval [CI] −0.19, 0.01), but a statistically significant decrease was seen at 12 months (predicted mean difference, −0.15; 95% CI −0.28, −0.02), and they had significantly lower symptoms of depression at both 6 and 12 months.
Tsitsi et al. (2017)	Cyprus and Greece	29/25 Age = 30–40 (mothers) Age = 37–43 (fathers)	Relaxation intervention Face to face	Cognitive behavior therapy	3 weeks	Anxiety	t0: Baseline t1: 3 weeks	Hamilton's Anxiety Scale (HAM‐A) Profile of Mood States Brief scale Tension/Anxiety, Depression/Dejection, Anger/Hostility	There was a statistically significant difference in the mean scores of the subjects in the intervention group on the HAM‐A scale between the T0 (14.67 ± 9.93) and T1 (11.70 ± 8.15) measurements. (*p* = 0.008) compared to the control group in which a borderline difference (16.00 ± 11.52 vs. 13.33 ± 8.38) was found (*p* =0.066).
Zhang et al. (2018)	China	22/22 Age Mean = 31	Solution‐focused brief therapy Face to face	Cognitive behavioral therapy	2 weeks	Anxiety Depression	t0: Baseline t1: 2 weeks	The Chinese version of the Brief Symptom Inventory (BSI‐18) = Distress, Anxiety, Depression	Parents in the SFBT group reported a significantly greater reduction in sub‐scores of somatization (*F*(1) = 6.52, *p* < 0.05), depression (*F*(1) = 19.85, *p* < 0.001), and anxiety (*F*(1) = 17.315, *p* < 0.001).

The parameters measured in the studies, measurement times, and measurement tools used are given in Table 1.

### 3.3 Psychosocial Interventions in Anxiety Management

**FIGURE 3:**
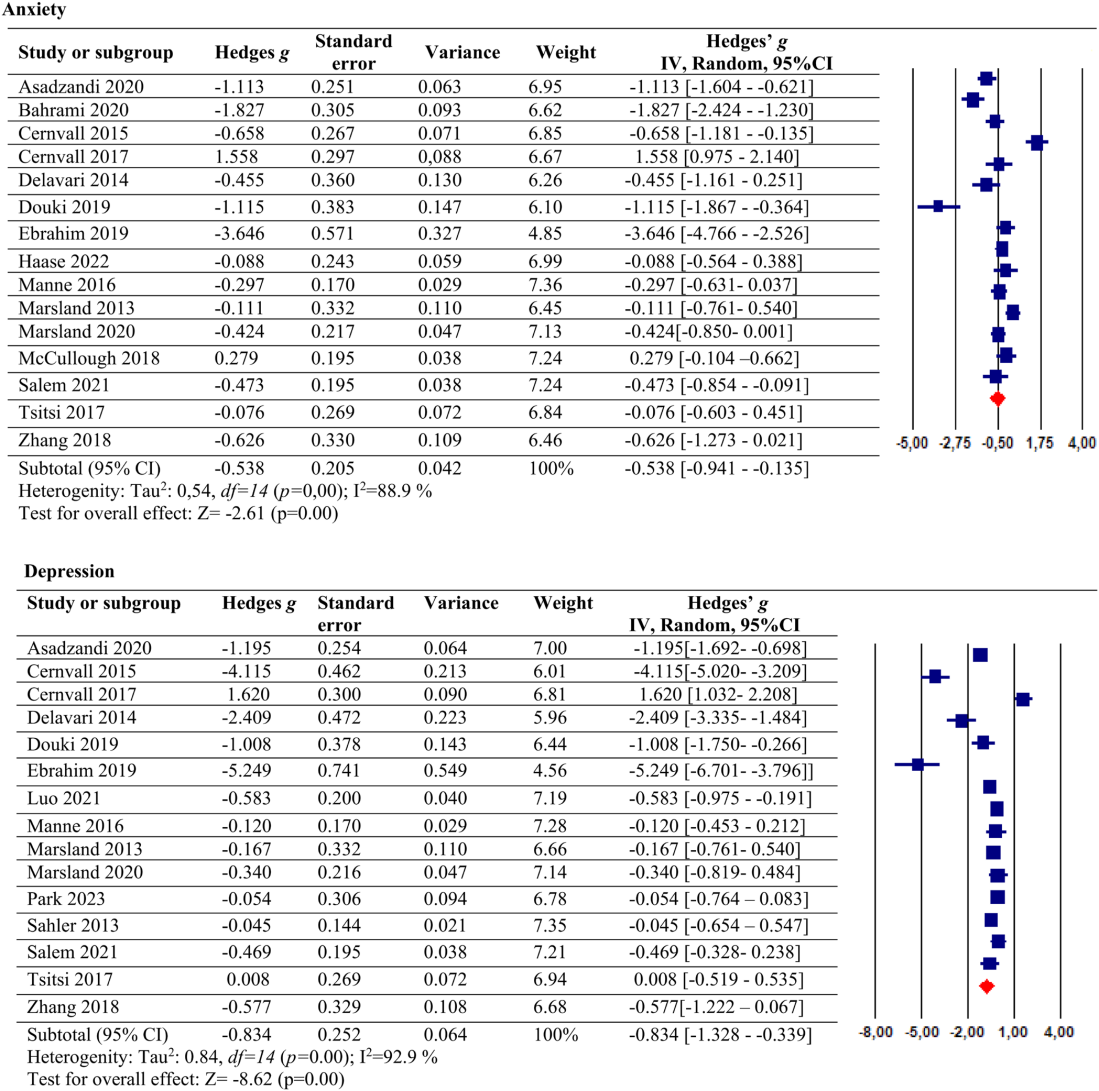
The effect of psychosocial interventions on anxiety and depression.

Figure 3 presents the efficacy of psychosocial interventions on anxiety. It was found in the study that psychosocial interventions had a moderate effect on anxiety relief (*g* = −0.538, 95% CI: [−0.941, −0.135], *p* < 0.05). However, a high level of overall heterogeneity was observed (*I* ^2^ = 88.9%, *p* = 0.00). The Egger's regression test (*p* = 0.13) and rank correlation test (*p* = 0.15) were not significant, suggesting no evidence of publication bias.

We evaluated the relationship between the effect sizes and the duration of intervention as predictors in the metaregression analysis. The results indicated that the duration of intervention was not a significant predictor of the psychosocial interventions in anxiety reduction (coefficient [SE], 0.0094 [0.0194]; *p* = 0.62).

### 3.4 Psychosocial Interventions in Depression Management

Figure 3 presents the efficacy of psychosocial interventions on depression. It was found in this meta‐analysis that psychosocial intervention had a large effect on depression relief in parents of children with cancer (*g* = −0.834, 95% CI: [−1.328, −0.339], *p* < 0.05). Heterogeneity results were obtained (*I* ^2^ = 92.9%, *p* = 0.00). To check the publishing bias, it was determined that for an effect size of 0, 276 studies were needed when the Rosenthal fail‐safe N according to the depression level of parents of children with cancer was examined. No publication bias was found.

When we evaluated with metaregression analysis whether psychosocial interventions are an important moderator affecting the effect size in reducing depression, we did not find a significant result (coefficient [SE], 0.0159 [0.0238]; *p* = 0.50).

### 3.5 Psychosocial Interventions in Quality‐Of‐Life Management

**FIGURE 4:**
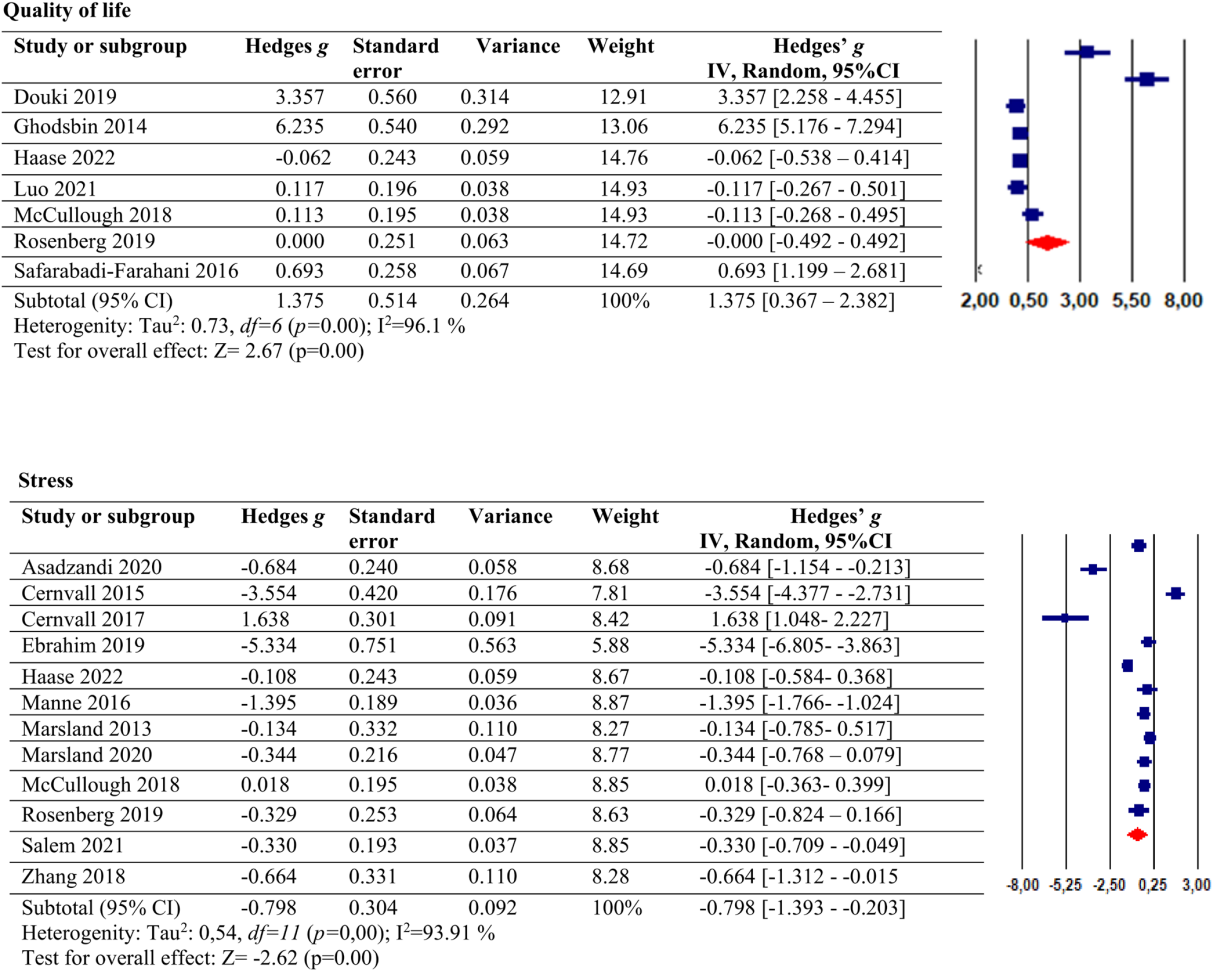
The effect of psychosocial interventions on the quality of life and stress.

Figure 4 presents the efficacy of psychosocial interventions on the quality of life. According to the results of this meta‐analysis, the psychosocial intervention had a large effect on the quality‐of‐life improvement (*g* = 1.375, 95% CI: [0.367, 2.382], *p* < 0.05). However, a high level of overall heterogeneity was observed (*I* ^2^ = 96%, *p* = 0.00). To check the publishing bias, the Fail‐Safe N revealed that 110 unpublished studies with null effects would need to be published to make the overall correlation nonsignificant.

We evaluated the relationship between the effect sizes and the duration of intervention as predictors in the metaregression analysis. The results indicated that the duration of intervention was not a significant predictor of the psychosocial interventions in the quality‐of‐life improvement (coefficient [SE], 0.0464 [0.1724]; *p* = 0.78).

### 3.6 Psychosocial Interventions in Stress Management

Figure 4 presents the efficacy of psychosocial interventions on stress. It was found in the study that psychosocial interventions had a moderate effect on stress (*g* = −0.798, 95% CI: [−1.393, −0.203], *p* < 0.05). However, a high level of overall heterogeneity was observed (*I* ^2^ = 88.9%, *p* = 0.00). The Egger's regression test (*p* = 0.13) and rank correlation test (*p* = 0.15) were not significant, suggesting no evidence of publication bias.

We evaluated the relationship between the effect sizes and the duration of intervention as predictors in the metaregression analysis. The results indicated that the duration of intervention was not a significant predictor of the psychosocial interventions in stress reduction (coefficient [SE], 0.0271 [0.0284]; *p* = 0.33).

### 3.7 Impact of Intervention Type

**FIGURE 5:**
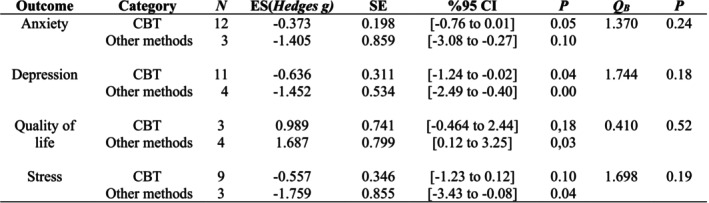
Effect sizes according to intervention type.

ANOVA was performed to examine whether the effect size varied according to the intervention type. Two types of intervention were defined in the study: cognitive behavioral therapies and other methods. There was no significant difference in terms of the effect size of cognitive behavioral therapies and other methods on anxiety, depression, quality of life, and stress (Figure 5).

### 3.8 Sensitivity Analysis

A leave‐one‐out sensitivity analysis was conducted to assess the robustness and reliability of the meta‐analysis findings for anxiety, depression, quality of life, and stress. This method systematically removes one study at a time to evaluate its influence on the overall pooled effect sizes (Borenstein et al. 2021; Higgins et al. 2019).

The analysis confirmed that no individual study had a disproportionate impact on the pooled effect sizes for any of the assessed outcomes, reinforcing the stability and reliability of the findings. These results strengthen confidence in the overall conclusions drawn from this meta‐analysis.

## 4 Discussion

Childhood cancers can affect both children and parents negatively. When children are diagnosed with cancer, it can cause a shock effect on parents, and they may feel intense fear and helplessness. Therefore, intervention practices are important in reducing psychosocial problems experienced by parents (Masulani‐Mwale et al. 2019; van Warmerdam et al. 2019). This meta‐analysis provides evidence on the effectiveness of psychosocial interventions applied to families of children diagnosed with cancer.

According to the results of this meta‐analysis, psychosocial interventions had a moderate effect on the relief of anxiety. Results showed that psychosocial intervention programs would be effective in reducing the anxiety of parents of children and adolescents followed up with a diagnosis of cancer. The results of this study are consistent with the results of the study conducted by Sánchez‐Egea et al. (2019) with parents of children diagnosed with cancer. However, in four meta‐analysis studies, psychosocial intervention programs for parents of children with chronic health problems (Sheng et al. 2023) and caregivers of patients with cancer (Cheng et al. 2022), but parents of children with cancer (Bautista et al. 2021; Eche et al. 2021) showed a low effect on reducing parents' anxiety levels. It was found that interventions to reduce psychosocial problems experienced by parents were effective in reducing anxiety, but not effective enough. In this direction, it is thought that the content of the intervention programs can be effective in reducing anxiety.

When we evaluate the effect of psychosocial interventions on depression in this study, it is seen that it has a great effect on alleviating depression. Like the results of the study we obtained, it was stated that the psychosocial intervention program for the parents of children followed up with cancer diagnosis was moderately (Eche et al. 2021) and highly (Bautista et al. 2021) effective in reducing the depression levels of parents. In addition, psychoeducation interventions given to the caregivers of people with cancer were moderately effective in reducing depression (Cheng et al. 2022). It was found that studies with similar results to the present research were characterized by including randomized controlled psychosocial interventions. Psychosocial interventions are thought to have important effects in alleviating the depression levels of parents. It is thought that there is a need for studies in which the effects of psychosocial interventions on depression are followed long term. Long‐term follow‐up will show that the initiatives are adopted by the parents and turned into a lifestyle.

Psychosocial interventions including randomized controlled trials were found to be effective in improving the quality of life of parents or family members. It was found that the interventions were highly effective in improving the quality of life of individuals. In a meta‐analysis study conducted for the parents of children followed up with a diagnosis of cancer, it was stated that interventions were highly effective in increasing the quality of life of parents (Bautista et al. 2021) and similar results were obtained with this study. It was stated that psychoeducational/psychosocial interventions for caregivers of cancer patients and children and adolescents followed up with a diagnosis of cancer had a low level of effect on increasing the quality of life of individuals (Cheng et al. 2022; Richter et al. 2015). The fact that psychosocial interventions were carried out in short periods and the small sample group may be effective in the low level of effect of the interventions on quality of life.

In this study, psychosocial interventions were moderately effective on stress. It has been reported that interventions for parents of children with cancer are highly effective in reducing stress (Bautista et al. 2021). Psychological treatments for family members of children with cancer were found to offer similar results to this study in reducing posttraumatic stress (Sánchez‐Egea et al. 2019).

When looking at the effect of psychosocial intervention type on anxiety, depression, quality of life, and stress, no significant difference was found between cognitive behavioral techniques and other psychosocial interventions. No evidence‐based study comparing cognitive behavioral techniques and other psychosocial interventions has been found in the literature. It is thought that this result will guide programs to be prepared for parents of children with cancer. Additionally, there appears to be a need for studies comparing cognitive behavioral interventions with other psychosocial interventions.

## 5 Strengths and Limitations

This meta‐analysis presents the current data of the last 10 years in which anxiety, depression, quality of life, and stress were evaluated separately. The fact that this study was conducted only on parents whose children are undergoing cancer treatment is one of its strengths in terms of the reliability of the results. Additionally, when the literature is examined, one of its strongest aspects is that it is an up‐to‐date meta‐analysis consisting of high‐quality randomized controlled studies evaluating the effectiveness of psychosocial interventions in parents of children receiving cancer treatment.

This study has several limitations. Many of the studies included in the meta‐analysis evaluated the effect of a single psychosocial intervention program on multiple outcomes (e.g., anxiety, depression, stress, quality of life); each outcome was assessed using distinct validated measurement tools. Therefore, although the same study data were used for different outcomes, using independent instruments reduces the risk of methodological bias. However, this approach may still introduce a dependency between effect sizes (Borenstein 2009). Future studies should consider techniques such as multivariate meta‐analysis to address the dependency among effect sizes. Another limitation of the study is the lack of long‐term follow‐up data on psychosocial interventions in most included studies. Although the meta‐analysis found significant improvements, particularly in depression and quality of life outcomes, it remains unclear to what extent these effects persist over time. Additional studies with extended follow‐up periods are needed to determine the long‐term sustainability of intervention effects.

Another limitation of this study is that it is based on articles published only in English, which we have determined as a research strategy. Heterogeneity was another challenge in this study, as differences in measurement tools and research quality contributed to variable effect sizes. To manage this, we performed subgroup analyses based on intervention type and conducted metaregression analysis based on intervention duration. However, some factors that may influence heterogeneity, such as sample characteristics and detailed characteristics of the control group, could not be assessed due to inconsistent reporting across studies. Future meta‐analyses with more standardized data reporting could further investigate potential moderators. Although we included only validated and widely used measurement tools for each outcome, we did not perform moderator analyses based on instrument type due to limited subgroup sample sizes. This may have contributed to unexplained heterogeneity and should be addressed in future studies.

Additionally, most of the psychosocial interventions analyzed were short‐term programs, which may limit assessing their long‐term effectiveness. More research is needed to evaluate whether short‐term improvements translate into sustained long‐term benefits. Another limitation of this study is that many of the included trials had small sample sizes, which may affect the generalizability of the findings. To mitigate this, we applied a random‐effects model to account for variability introduced by small studies. Finally, although subgroup analyses were conducted to compare the effects of different intervention types, we did not perform interaction analyses between interventions such as those possible through network meta‐analysis. Future research should consider network meta‐analytic approaches to better examine the comparative effectiveness and potential interactions among various psychosocial strategies.

## 6 Conclusion

Psychosocial interventions play an important role in reducing anxiety, depression, and stress of parents and increasing their quality of life. This study provides clinical evidence for the effectiveness of psychosocial intervention programs for parents of children undergoing cancer treatment. The findings of this study indicated that the impact of psychosocial interventions was characterized by medium to high levels of anxiety, depression, quality of life, and stress. The efficacy of psychosocial interventions developed for parents may be subject to variation. It is hypothesized that by incorporating psychological well‐being, acceptance, and the mourning process into the comprehensive design of these intervention programs, the efficacy of the programs will be further enhanced.

It is recommended that future studies include longer follow‐up periods and larger sample sizes to enhance short‐ and long‐term psychosocial health outcomes for parents. Future studies should prioritize the effectiveness of follow‐up programs and alternative intervention strategies designed to strengthen parental psychological well‐being. Furthermore, intervention programs should provide more information to facilitate reproducibility and clinical dissemination in other cultures.

## 7 Relevance for Clinical Practice

This study showed that psychosocial interventions for parents of children with cancer were effective in reducing stress, anxiety, and depression and improving quality of life. However, the lack of a significant relationship between the duration and effectiveness of the interventions emphasizes the importance of focusing on the content and quality of interventions rather than their duration in clinical practice. In this context, various strategies can be suggested, such as brief and intensive interventions in which pediatric nurses take an active role, individualized plans, and online and technological support tools. Pediatric nurses provide psychosocial support to parents, making a critical contribution to regular follow‐up, early intervention, and collaboration processes of multidisciplinary teams. Utilizing the expertise of pediatric nurses to improve parents' stress‐coping skills and improve quality of life can strengthen the impact of interventions. Such strategic approaches will play an essential role in maximizing the effectiveness of interventions and improving parents' experiences during the care process.

## Author Contributions

All authors contributed to the study conception and design; material preparation, data collection, and analysis; and read and approved the final manuscript.

## Conflicts of Interest

The authors declare no conflicts of interest.

## Supporting information


File S1.

File S2.

File S3.

File S4.

## Data Availability

Data sharing not applicable to this article as no datasets were generated or analysed during the current study.
