# Supplementary material for: Effectiveness of Psychosocial Interventions on Stress, Anxiety, Depression, and Quality of Life in Parents of Children, Adolescents, and Young Adults With Cancer: A Meta‐Analysis of RCTs
Source: Nurs Health Sci. 2025 Jun 10;27(2):e70156. doi: 10.1111/nhs.70156 (PMC12151627; doi:10.1111/nhs.70156)
Supplement: Supplementary file 2 — File S2. [file NHS-27-e70156-s002.docx]

| **Main Concept** | **#** | **Research strategy** |
| --- | --- | --- |
| A  (Child, Adolescent, Young Adults) | **#1** | ("adolescent" OR "teen" OR "youth" OR "young adult" OR "young adulthood" OR "young people" OR "pediatric oncology" OR "children" OR "child): ti,ab |
| B  (Cancer) | **#2** | ("oncolog" OR "cancer" OR "malignan" OR "neoplas" OR "tumor" OR "leukemia" OR "sarcoma" OR "caricinoma"):ti,ab |
| C  (Parents, family) | **#3** | ("family" OR "parents" OR "caregivers"): ti,ab |
| D  (Interventions) | **#4** | ("intervention*" OR "prevent" OR "social" OR "psycho*" OR "support" OR "psychosocial interventions" OR "intervention for parents" OR "CBT" OR "cognitive behavioral therapy" OR "resilience" OR "coping" OR "stress management" OR "problem-solving therapy"): ti,ab |
| E  (Randomized controlled trial) | **#5** | (randomized controlled trial):pt OR (randomized controlled trial): ti,ab |
| **Limitations: (Language [English] AND publication years [2013-2024])**  **1 AND 2 AND 3 AND 4 AND 5**  **Combined search results: 717** | | |

**Literature Search Strings**

**Cochrane (April 30 Th, 2024)**

| **Main Concept** | **#** | **Research strategy** |
| --- | --- | --- |
| A  (Child, Adolescent, Young Adults) | **#1** | MM ("Child" OR "Adolescent" OR "Young Adult" OR "Cancer Patients") OR AB ("adolescent" OR "teen" OR "youth" OR "young adult" OR "young adulthood" OR "pediatric oncology" OR "children"" OR "child") OR TI ("adolescent" OR "teen" OR "youth" OR "young adult" OR "young adulthood" OR "pediatric oncology" OR "children"" OR "child") |
| B  (Cancer) | **#2** | MM ("Neoplasms" OR "Oncology") OR TI ("oncolog" OR "cancer" OR "malignan" OR "neoplas" OR "tumor" OR "leukemia" OR "sarcoma" OR "caricinoma") OR AB ("oncolog" OR "cancer" OR "malignan" OR "neoplas" OR "tumor" OR "leukemia" OR "sarcoma" OR "caricinoma") |
| C  (Parents, family) | **#3** | TI (family OR parents OR caregivers OR parents of children with cancer OR family of children with cancer) OR AB (family OR parents OR caregivers OR parents of children with cancer OR family of children with cancer) |
| D  (Interventions) | **#4** | TI ("intervention" OR "prevent" OR "social" OR " psychosocial " OR "support" OR "psychosocial interventions" OR "intervention for parents") OR AB (“intervention" OR "prevent" OR "social" OR " psychosocial" OR "support" OR "psychosocial interventions" OR intervention for parents" OR "CBT" OR "cognitive behavioral therapy" OR "resilience" OR "coping" OR "stress management" OR "problem-solving therapy) |
| E  (Randomized controlled trial) | **#5** | MM "randomized controlled trial" OR PT ("controlled clinical trial" OR "randomized controlled trial") OR TI ("controlled clinical trial" OR "randomized controlled trial") OR AB ("controlled clinical trial" OR "randomized controlled trial") |
| **Limitations: (2013-2024) AND (English)**  **1 AND 2 AND 3 AND 4 AND 5**  **Combined search results: 323** | | |

**MEDLINE (April 30 Th, 2024)**

| **Main Concept** | **#** | **Research strategy** |
| --- | --- | --- |
| A  (Child, Adolescent, Young Adults) | **#1** | ti("adolescent" OR "teen" OR "youth" OR "young adult" OR "young adulthood" OR "pediatric oncology" OR "children" OR "child") OR ab("adolescent" OR "teen" OR "youth" OR "young adult" OR "young adulthood" OR "pediatric oncology" OR "children" OR "child") |
| B  (Cancer) | **#2** | ti("oncolog" OR cancer* OR "malignan" OR "neoplasm" OR "tumor" OR "leukemia" OR "sarcoma" OR "caricinoma") OR ab("oncolog" OR cancer* OR "malignan" OR "neoplasm" OR "tumor" OR "leukemia" OR "sarcoma" OR "caricinoma") |
| C  (Parents, family) | **#3** | ti(family OR parents OR caregivers OR parents of children with cancer OR family of children with cancer) OR ab(family OR parents OR caregivers OR parents of children with cancer OR family of children with cancer) |
| D  (Interventions) | **#4** | ti(intervention* OR prevent OR social OR psycho* OR support OR psychosocial interventions OR intervention for parents) OR ab( intervention* OR prevent OR social OR psycho* OR support OR psychosocial interventions OR intervention for parents OR CBT OR cognitive behavioral therapy OR resilience OR coping OR stress management OR problem-solving therapy) |
| E  (Randomized controlled trial) | **#6** | ti("controlled clinical trial" OR "randomized controlled trial") OR ab("controlled clinical trial" OR "randomized controlled trial") |
| **Limitations: (Language [English] AND publication years [2013-2024])**  **1 AND 2 AND 3 AND 4 AND 5 AND 6**  **Combined search results: 4142** | | |

**Proquest (April 30 Th, 2024)**

**PubMED** **(April 30 Th, 2024)**

| **Main Concept** | **#** | **Research strategy** |
| --- | --- | --- |
| A  (Child, Adolescent, Young Adults) | **#1** | ("child"[MeSH Terms] OR "pediatrics"[MeSH Terms]) [Title/ Abstract] |
| B  (Cancer) | **#2** | ("cancer"[All Fields] OR "neoplasms"[MeSH Terms] OR childhood cancer"[All Fields] OR "children with cancer"[All Fields] OR "paediatric oncology"[All Fields] OR "paediatric cancer"[All Fields] OR "childhood cancer survivors"[All Fields] OR "AYA survivors"[All Fields] OR "childhood cancer survivors"[All Fields] OR "pediatric cancer survivors"[All Fields] OR "adolescent cancer survivors"[All Fields]) ) [Title/ Abstract] |
| C  (Parents, family) | **#3** | ("family"[MeSH Terms] OR "parents"[MeSH Terms] OR "caregivers"[MeSH Terms] OR "parents of children with cancer"[All Fields] OR "family"[MeSH Terms]) [Title/ Abstract] |
| D  (Interventions) | **#4** | ("interventions"[All Fields] OR Prevent OR social OR psycho* OR support OR "psychosocial interventions" OR CBT OR "cognitive behavioral therapy" OR resilience OR coping OR "stress management" OR "problem-solving therapy" [Title/ Abstract] |
| E  (Randomized controlled trial) | **#5** | (randomized controlled trial*[MeSH Terms]) OR  (controlled clinical trial*[Publication Type] OR randomized controlled trial*[Publication Type]) OR (controlled clinical trial*[Title/Abstract] OR randomized controlled trial* |
| A+B+C+D+E | **#6** | 1 AND 2 AND 3 AND 4 AND 5 |
| A+B+C+D+E | **#7** | Limit 6 to (English) |
| A+B+C+D+E | **#8** | Limit 7 to (2013-2024) [Publication Years] |
| **6 AND 7 AND 9**  **Combined search results: 630** | | |

**Science Direct (April 30 Th, 2024)**

| **Main Concept** | **#** | **Research strategy** |
| --- | --- | --- |
| A  (Child, Adolescent, Young Adults) | **#1** | pediatric OR child (Title, abstract or author-specified keywords) |
| B  (Cancer) | **#2** | cancer OR childhood cancer (Title, abstract or author-specified keywords) |
| C  (Parents, family) | **#3** | family OR parents (Title, abstract or author-specified keywords) |
| D  (Interventions) | **#4** | intervention (Title, abstract or author-specified keywords) |
| E  (Randomized controlled trial) | **#5** | randomized controlled trial (Title, abstract or author-specified keywords) |
| **Limitations: 2013-2024**  **1 AND 2 AND 3 AND 4 AND 5**  **Combined search results: 3349** | | |

**Scopus (April 30 Th, 2024)**

| **Main Concept** | **#** | **Research strategy** |
| --- | --- | --- |
| A  (Child, Adolescent, Young Adults) | **#1** | TITLE-ABS-KEY (child OR children OR paediatric OR pediatric) |
| B  (Cancer) | **#2** | TITLE-ABS-KEY (cancer OR neoplasm OR childhood AND cancer OR children AND with AND cancer OR paediatric AND oncology OR paediatric AND cancer OR childhood AND cancer OR pediatric AND cancer AND survivors OR adolescent AND cancer) |
| C  (Parents, family) | **#3** | TITLE-ABS-KEY (family OR parents OR caregivers OR parents AND of AND children AND with AND cancer OR family AND of AND children AND with AND cancer) |
| D  (Interventions) | **#4** | TITLE-ABS-KEY (intervention* OR prevent OR social OR psycho* OR support OR psychosocial AND interventions OR intervention OR CBT OR cognitive behavioral therapy OR resilience OR coping OR stress management OR problem-solving therapy) |
| E  (Randomized controlled trial) | **#5** | TITLE-ABS-KEY (controlled AND clinical AND trial or randomized AND controlled AND trial) |
| **No limitations: Date, Language**  **1 AND 2 AND 3 AND 4 AND 5**  **Combined search results: 14** | | |

**Web Of Science (April 30 Th, 2024)**

| **Main Concept** | **#** | **Research strategy** |
| --- | --- | --- |
| A  (Child, Adolescent, Young Adults) | **#1** | (Child OR children OR paediatric OR pediatric). ti,ab |
| B  (Cancer) | **#2** | (Cancer OR neoplasm OR oncology). ti,ab |
| C  (Parents, family) | **#3** | (Family OR parents OR caregivers). ti,ab |
| D  (Interventions) | **#4** | (intervention* OR prevent OR social OR psycho* OR support OR psychosocial OR CBT OR cognitive behavioral therapy OR resilience OR coping OR stress management OR problem-solving therapy). ti, ab |
| E  (Randomized controlled trial) | **#6** | (Randomized controlled trial). ti, ab |
| A+B+C+D+E | **#7** | 1 AND 2 AND 3 AND 4 AND 5 AND 6 |
| A + B+C+D+E | **#8** | Limit 7 to [English (Languages)] |
| A + B+C+D+E | **#9** | Limit 8 to [2013-2024 (Year Published)] |
| **7 AND 8 AND 9**  **Combined search results: 152** | | |
