# Supplementary material for: Effectiveness of Psychosocial Interventions on Stress, Anxiety, Depression, and Quality of Life in Parents of Children, Adolescents, and Young Adults With Cancer: A Meta‐Analysis of RCTs
Source: Nurs Health Sci. 2025 Jun 10;27(2):e70156. doi: 10.1111/nhs.70156 (PMC12151627; doi:10.1111/nhs.70156)
Supplement: Supplementary file 3 — File S3. [file NHS-27-e70156-s001.docx]

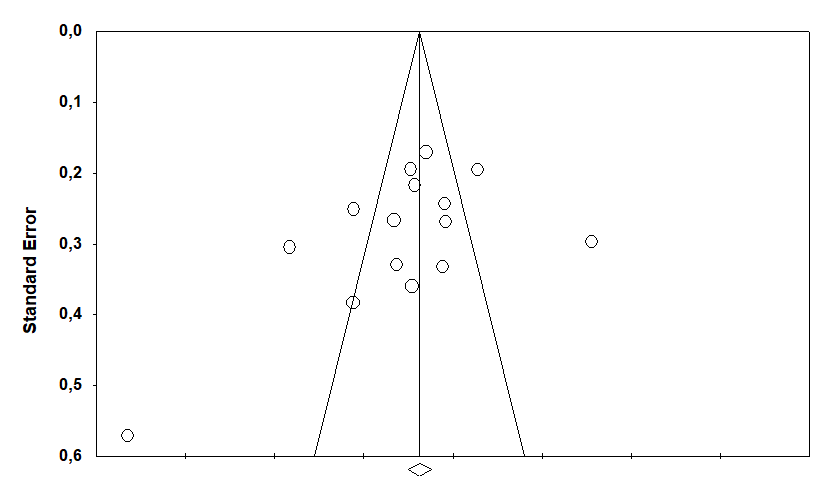


**Figure 1.** Funnel plot for parents’ anxiety


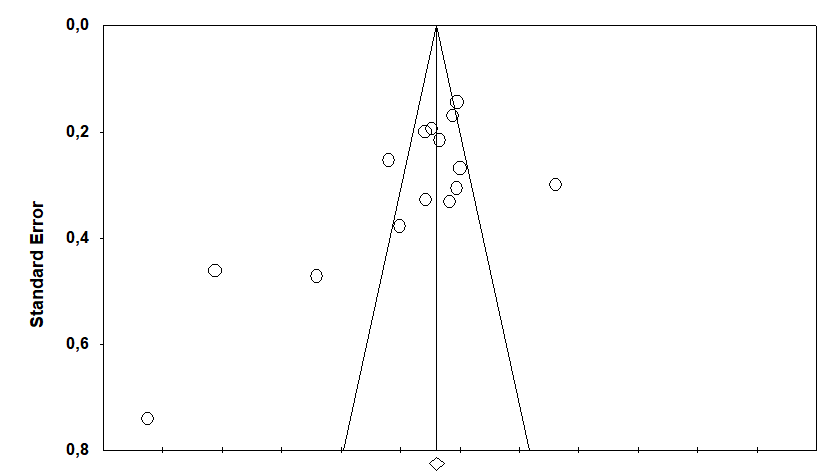


**Figure 2.** Funnel plot for parent’s depression


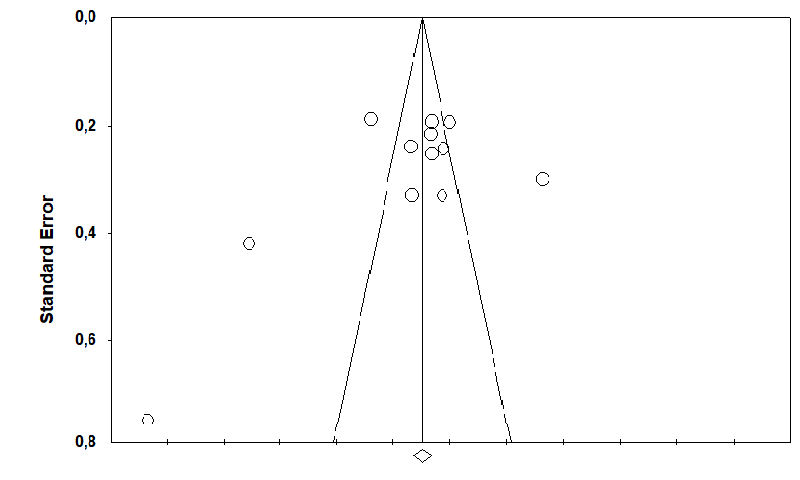


**Figure 3**. Funnel plot for parents’ stress
