# Supplementary material for: Effectiveness of Psychosocial Interventions on Stress, Anxiety, Depression, and Quality of Life in Parents of Children, Adolescents, and Young Adults With Cancer: A Meta‐Analysis of RCTs
Source: Nurs Health Sci. 2025 Jun 10;27(2):e70156. doi: 10.1111/nhs.70156 (PMC12151627; doi:10.1111/nhs.70156)
Supplement: Supplementary file 4 — File S4. [file NHS-27-e70156-s004.docx]

| **List of Excluded Studies** | | | | |
| --- | --- | --- | --- | --- |
| **No** | **Author, year** | **Title of the study** | **References** | **Reasons for exclusion** |
| 1 | Asadi Noughabi et al., 2015 | Effect of Instructing Care Program Through Group Discussion on the Quality of Life of the Parents of the Children Afflicted with Leukemia | Asadi Noughabi F, Iranpoor D, Yousefi H, Abrakht H, Ghani Dehkordi F. Effect of Instructing Care Program Through Group Discussion on the Quality of Life of the Parents of the Children Afflicted With Leukemia. Glob J Health Sci. 2015 Oct 20;8(5):197-204. doi: 10.5539/gjhs.v8n5p197. PMID: 26652069; PMCID: PMC4877215. | A quasi-experimental study |
| 2 | Asadzandi et al., 2021 | Effect of spiritual care on the care burden of families of children with cancer: a randomized controlled trial' | Asadzandi, M., S. S. Farahany, H. Abolghasemy, M. Saberi, and A. Ebadi. 2021. 'Effect of spiritual care on the care burden of families of children with cancer: a randomized controlled trial', Family Medicine and Primary Care Review, 23: 279-83. | In this study, only the "care burden" outcome was examined. |
| 3 | Baron Nelson et al., 2018 | Adding a parent to the brain tumour team: Evaluating a peer support intervention for parents of children with brain tumours | Baron Nelson, M., Riley, K., & Arellano, K. (2018). Adding a parent to the brain tumour team: Evaluating a peer support intervention for parents of children with brain tumours. Journal of Pediatric Oncology Nursing, 35(3), 218-228. | This was a mixed-methods, cross-sectional study |
| 4 | Barrea 2014 | Reduction of Anxiety Levels in Parents and Siblings of Children with Cancer After Sibling Participation in A Psychosocial Group Intervention: A Randomized Controlled Trial | Barrera, M., A. Rokeach, K. Hancock, F. Schulte, E. Atenafu, and P. Nathan. 2014. 'Reduction of Anxiety Levels in Parents and Siblings of Children with Cancer After Sibling Participation in A Psychosocial Group Intervention: A Randomized Controlled Trial', Pediatric Blood & Cancer, 61: S161-S61. | This study is siblings-centered psychosocial intervention |
| 5 | Barrea 2018 | A randomized controlled trial of a group intervention for siblings of children with cancer: Changes in symptoms of anxiety in siblings and caregivers | Barrera, M., E. G. Atenafu, F. Schulte, P. C. Nathan, K. Hancock, and A. Saleh. 2018. 'A randomized controlled trial of a group intervention for siblings of children with cancer: Changes in symptoms of anxiety in siblings and caregivers', Psycho-Oncology, 27: 1629-34. | This study is siblings-centered psychosocial intervention |
| 6 | Barrea 2020 | Quality of life in pediatric oncology patients, caregivers and siblings after psychosocial screening: a randomized controlled trial | Barrera, M., Hancock, K., Atenafu, E., Alexander, S., Solomon, A., Desjardins, L., ... & Mills, D. 2020. 'Quality of life in pediatric oncology patients, caregivers and siblings after psychosocial screening: a randomized controlled trial', Supportive Care in Cancer, 28: 3659-68. | This study is siblings-centered psychosocial intervention |
| 7 | Beheshtipour et al., 2016 | The effect of educational-spiritual intervention on the burnout of the parents of school age children with cancer: A randomized controlled clinical trial' | Beheshtipour, N., P. Nasirpour, S. Yektatalab, M. Karimi, and N. Zare. 2016. 'The effect of educational-spiritual intervention on the burnout of the parents of school age children with cancer: A randomized controlled clinical trial', International Journal of Community Based Nursing and Midwifery, 4: 90-97. | In this study, only the "burnout" outcome was examined. |
| 8 | Bidstrup et al., 2023 | Effects on Pediatric Cancer Survivors: The FAMily-Oriented Support (FAMOS) Randomized Controlled Trial | Bidstrup, P. E., H. Salem, E. W. Andersen, K. Schmiegelow, S. Rosthoj, P. S. Wehner, H. Hasle, S. O. Dalton, C. Johansen, and A. E. Kazak. 2023. 'Effects on Pediatric Cancer Survivors: The FAMily-Oriented Support (FAMOS) Randomized Controlled Trial', Journal of Pediatric Psychology, 48: 29-38. | Inappropriate sample |
| 9 | Borjalilu et al., 2016 | Spiritual Care Training for Mothers of Children with Cancer: Effects on Quality of Care and Mental Health of Caregivers | Borjalilu, S., Shahidi, S., Mazaheri, M. A., & Emami, A. H. (2016). Spiritual care training for mothers of children with cancer: Effects on quality of care and mental health of caregivers. Asian Pac J Cancer Prev, 17(2), 545-552. | a quasi-experimental study |
| 10 | Dolgin et al., 2021 | Responsivity to Problem-Solving Skills Training in Mothers of Children with Cancer | Dolgin, M. J., K. A. Devine, D. Tzur-Bitan, M. A. Askins, D. L. Fairclough, E. R. Katz, R. B. Noll, S. Phipps, and O. J. Z. Sahler. 2021. 'Responsivity to Problem-Solving Skills Training in Mothers of Children with Cancer', Journal of Pediatric Psychology, 46: 413-21. | These findings are from secondary outcome of a RCT (Sahler et al., 2013). |
| 11 | Fedele et al., 2013 | Impact of a parent-based interdisciplinary intervention for mothers on adjustment in children newly diagnosed with cancer | Fedele, D. A., S. E. Hullmann, M. Chaffin, C. Kenner, M. J. Fisher, K. Kirk, A. R. Eddington, S. Phipps, R. Y. McNall-Knapp, and L. L. Mullins. 2013. 'Impact of a parent-based interdisciplinary intervention for mothers on adjustment in children newly diagnosed with cancer', J Pediatr Psychol, 38: 531-40. | The study has been conducted for children |
| 12 | Feraco et al., 2017 | Tumor Talk and Child Well-Being: Perceptions of “Good” and “Bad” News Among Parents of Children With Advanced Cancer | Feraco, Angela M., Veronica Dussel, Liliana Orellana, Tammy I. Kang, J. Russell Geyer, Abby R. Rosenberg, Chris Feudtner, and Joanne Wolfe. 2017. 'Tumor Talk and Child Well-Being: Perceptions of “Good” and “Bad” News Among Parents of Children With Advanced Cancer', Journal of Pain and Symptom Management, 53: 833-41. | Inappropriate outcomes were evaluated. |
| 13 | Gårdling et al., 2018 | Impact of Age-appropriate Preparations for Children with Cancer Undergoing Radiotherapy on Parents and Family Functioning, Parents' Anxiety and Hospital Costs–A Feasibility Study | Gårdling, J., Törnqvist, E., Månsson, M. E., & Hallström, I. K. (2018). Impact of Age-appropriate Preparations for Children with Cancer Undergoing Radiotherapy on Parents and Family Functioning, Parents' Anxiety and Hospital Costs–A Feasibility Study. Journal of Pediatric Nursing, 43, e51-e58. | Quasi-experimental controlled clinical trial-Feasibility |
| 14 | Gouveia et al., 2017 | Comparing two types of perspective taking as strategies for detecting distress amongst parents of children with cancer: A randomised trial | Gouveia, L., A. Janvier, F. Dupuis, M. Duval, and S. Sultan. 2017. 'Comparing two types of perspective taking as strategies for detecting distress amongst parents of children with cancer: A randomised trial', PLoS One, 12: e0175342. | This study was conducted to compare two perspective-taking strategies to understand parents. |
| 15 | Hauken et al., 2018 | The Effects on Children's Anxiety and Quality of Life of a Psychoeducational Program for Families Living With Parental Cancer and Their Network: A Randomized Controlled Trial Study | Hauken, M. A., M. Pereira, and M. Senneseth. 2018. 'The Effects on Children's Anxiety and Quality of Life of a Psychoeducational Program for Families Living With Parental Cancer and Their Network: A Randomized Controlled Trial Study', Cancer Nursing, 41: 473-83. | Inappropriate sample |
| 16 | Hoseinzadeh et al., 2019 | Effect of Resilience-based Group Therapy Intervention on Coping in Mothers of Children with Cancer: A Randomized Clinical Trial | Hoseinzadeh, F., M. Radfar, F. Moghaddamtabrizi, and H. Khalkhali. 2019. 'Effect of Resilience-based Group Therapy Intervention on Coping in Mothers of Children with Cancer: A Randomized Clinical Trial', Iranian Journal of Nursing and Midwifery Research, 24: 291-95. | In this study, only the "coping" outcome was examined. |
| 17 | Jamali et al., 2019 | Effect of peer education on the resilience of mothers of children with leukaemia: A clinical trial | Jamali, A., Ghaljaei, F., Keikhaei, A., & Jalalodini, A. (2019). Effect of peer education on the resilience of mothers of children with leukemia: A clinical trial. Medical-Surgical Nursing Journal, 8(2) | In this study, only the "resilience" outcome was examined. |
| 18 | Jin et al., 2023 | Feasibility and preliminary effects of acceptance and commitment therapy on reducing psychological distress and improving the quality of life of the parents of children with cancer: A pilot randomised controlled trial' | Jin, X. H., H. Y. Li, Y. Y. Chong, K. F. Mann, W. Y. Yao, and C. L. Wong. 2023. 'Feasibility and preliminary effects of acceptance and commitment therapy on reducing psychological distress and improving the quality of life of the parents of children with cancer: A pilot randomised controlled trial', Psycho-Oncology, 32: 165-69. | This study is a feasibility and acceptability study (inappropriate method) |
| 19 | Kim et al., 2015 | The influence of family management style on psychosocial problems of childhood cancer survivors in Korea | Kim, Dong Hee, and Yeo Jin Im. 2015. 'The influence of family management style on psychosocial problems of childhood cancer survivors in Korea', European Journal of Oncology Nursing, 19: 107-12. | It was conducted to describe psychosocial problems (Inappropriate method) |
| 20 | Lamanna et al., 2013 | A brief problem solving intervention for caregivers of children with cancer. | Lamanna, J. D. 2013. 'A brief problem solving intervention for caregivers of children with cancer.', Dissertation Abstracts International: Section B: The Sciences and Engineering, 73(9-B(E)). | Insufficient data |
| 21 | Mohammed et al., 2018 | I didn't want to be in charge and yet I was": Bereaved caregivers' accounts of providing home care for family members with advanced cancer | Mohammed, S., N. Swami, A. Pope, G. Rodin, B. Hannon, R. Nissim, S. Hales, and C. Zimmermann. 2018. '"I didn't want to be in charge and yet I was": Bereaved caregivers' accounts of providing home care for family members with advanced cancer', Psycho-Oncology, 27: 1229-36. | Qualitative study |
| 22 | Muscara et al., 2020 | Effect of a Videoconference-Based Online Group Intervention for Traumatic Stress in Parents of Children With Life-threatening Illness: A Randomized Clinical Trial | Muscara, F., M. C. McCarthy, M. Rayner, J. M. Nicholson, A. Dimovski, L. McMillan, S. J. C. Hearps, J. Yamada, K. Burke, R. Walser, and V. A. Anderson. 2020. 'Effect of a Videoconference-Based Online Group Intervention for Traumatic Stress in Parents of Children With Life-threatening Illness: A Randomized Clinical Trial', JAMA Netw Open, 3: e208507. | Inappropriate sample, this study about parents of children with life-threatening illness |
| 23 | Needle et al., 2022 | Effect of the Family-Centered Advance Care Planning for Teens with Cancer Intervention on Sustainability of Congruence About End-of-Life Treatment Preferences a Randomized Clinical Trial | Needle, J. S., S. Friebert, J. D. Thompkins, D. H. Grossoehme, J. N. Baker, J. J. Jiang, J. C. Wang, and M. E. Lyon. 2022. 'Effect of the Family-Centered Advance Care Planning for Teens with Cancer Intervention on Sustainability of Congruence About End-of-Life Treatment Preferences a Randomized Clinical Trial', Jama Network Open, 5. | Inappropriate outcomes were evaluated. |
| 24 | Phipps, 2020 | In-person vs. web-based administration of a problem-solving skills intervention for parents of children with cancer: Report of a randomized noninferiority trial | Phipps, S., Fairclough, D. L., Noll, R. B., Devine, K. A., Dolgin, M. J., Schepers, S. A., ... & Sahler, O. J. Z. 2020. 'n-person vs. web-based administration of a problem-solving skills intervention for parents of children with cancer: Report of a randomized noninferiority trial.', EClinicalMedicine, 24. | Inappropriate outcomes were evaluated. |
| 25 | Pouraboli et al., 2019 | The Effect of Relaxation Techniques on Anxiety, Fatigue and Sleep Quality of Parents of Children with Leukemia under Chemotherapy in South East Iran | Pouraboli, B., Z. Poodineh, and Y. Jahani. 2019. 'The Effect of Relaxation Techniques on Anxiety, Fatigue and Sleep Quality of Parents of Children with Leukemia under Chemotherapy in South East Iran', Asian Pac J Cancer Prev, 20: 2903-08. | a quasi-experimental study |
| 26 | Rayner et al., 2016 | Take A Breath: study protocol for a randomized controlled trial of an online group intervention to reduce traumatic stress in parents of children with a life-threatening illness or injury | Rayner, M., F. Muscara, A. Dimovski, M. C. McCarthy, J. Yamada, V. A. Anderson, K. Burke, R. Walser, and J. M. Nicholson. 2016. 'Take A Breath: study protocol for a randomized controlled trial of an online group intervention to reduce traumatic stress in parents of children with a life-threatening illness or injury', BMC Psychiatry, 16: 169. | This study is protocol for a randomized controlled trial |
| 27 | Ringner 2023 | Effects of Person-Centered Information for Parents of Children with Cancer (the PIFBO Study): A Randomized Controlled Trial | Ringner, A., M. Bjork, and C. Olsson. 2023. 'Effects of Person-Centered Information for Parents of Children with Cancer (the PIFBO Study): A Randomized Controlled Trial', Journal of Pediatric Hematology-Oncology Nursing. | Inappropriate intervention |
| 28 | Shakiba 2020 | The Effect of Cognitive-Emotional Intervention on Growth and Posttraumatic Stress in Mothers of Children With Cancer: A Randomized Clinical Trial | Shakiba, M., A. Latifi, and A. Navidian. 2020. 'The Effect of Cognitive-Emotional Intervention on Growth and Posttraumatic Stress in Mothers of Children With Cancer: A Randomized Clinical Trial', Journal of Pediatric Hematology Oncology, 42: 118-25. | Inappropriate outcomes were evaluated. |
| 29 | Thiblin et al., 2023 | Internet-administered, low-intensity cognitive behavioral therapy for parents of children treated for cancer: A feasibility trial (ENGAGE) | Thiblin, E., J. Woodford, C. Reuther, J. Lundgren, N. Lutvica, and L. von Essen. 2023. 'Internet-administered, low-intensity cognitive behavioral therapy for parents of children treated for cancer: A feasibility trial (ENGAGE)', Cancer Medicine, 12: 6225-43. | Inappropriate method, pilot feasibility trials |
| 30 | Tsitsi et al., 2020 | rogressive Muscle Relaxation and guided imagery as techniques to enhance the way of coping of parents of children with malignancies: Findings from a randomized controlled trial | Tsitsi, T., Raftopoulos, V., Papastavrou, E., & Charalambous, A. (2020). Progressive Muscle Relaxation and guided imagery as techniques to enhance the way of coping of parents of children with malignancies: Findings from a randomized controlled trial. European Journal of Oncology Nursing, 46, 101718. | These findings are from the secondary outcome of a RCT. |
| 31 | van Dijk‐Lokkart, et al., 2016 | Effects of a combined physical and psychosocial intervention program for childhood cancer patients on quality of life and psychosocial functioning: results of the QLIM randomized clinical trial | van Dijk‐Lokkart, E. M., Braam, K. I., van Dulmen‐den Broeder, E., Kaspers, G. J., Takken, T., Grootenhuis, M. A., ... & Huisman, J. (2016). Effects of a combined physical and psychosocial intervention program for childhood cancer patients on quality of life and psychosocial functioning: results of the QLIM randomized clinical trial. Psycho‐Oncology, 25(7), 815-822. | The study has been conducted for children |
| 32 | Wakefield et al., 2015 | Online parent-targeted cognitive-behavioural therapy intervention to improve quality of life in families of young cancer survivors: study protocol for a randomised controlled trial' | Wakefield, C. E., U. M. Sansom-Daly, B. C. McGill, M. McCarthy, A. Girgis, M. Grootenhuis, B. Barton, P. Patterson, M. Osborn, C. Lowe, A. Anazodo, G. Miles, and R. J. Cohn. 2015. 'Online parent-targeted cognitive-behavioural therapy intervention to improve quality of life in families of young cancer survivors: study protocol for a randomised controlled trial', Trials, 16: 153. | This study is a multi-site, randomised controlled trial (RCT) to assess the feasibility and efficacy. |
| 33 | Williams et al., 2016 | Addressing behavioral impacts of childhood leukemia: A feasibility pilot randomized controlled trial of a group videoconferencing parenting intervention | Williams, Lauren K., Maria C. McCarthy, Kylie Burke, Vicki Anderson, and Nicole Rinehart. 2016. 'Addressing behavioral impacts of childhood leukemia: A feasibility pilot randomized controlled trial of a group videoconferencing parenting intervention', European Journal of Oncology Nursing, 24: 61-69. | A feasibility pilot randomized controlled trial |
| 34 | Yi-Frazier et al., 2017 | Promoting Resilience in Stress Management for Parents (PRISM-P): An intervention for caregivers of youth with serious illness. | Yi-Frazier, J. P., Fladeboe, K., Klein, V., Eaton, L., Wharton, C., McCauley, E., & Rosenberg, A. R. (2017). Promoting Resilience in Stress Management for Parents (PRISM-P): An intervention for caregivers of youth with serious illness. Families, Systems, & Health, 35(3), 341. | This study is specific to families of children with both diabetes and cancer diagnosis. |
